# Supplementary material for: Self-assembled nematic colloidal motors powered by light
Source: Nat Commun. 2018 Nov 28;9:5040. doi: 10.1038/s41467-018-07518-x (PMC6261955; doi:10.1038/s41467-018-07518-x)
Supplement: Supplementary file 1 — Description of Additional Supplementary Files [file 41467_2018_7518_MOESM1_ESM.pdf]

Description of Additional Supplementary Files

File Name: Supplementary Movie 1
Description: Polarizing optical textures of a platelet spinning under continuous exposure to blue excitation light with Pe ǁ n0 at the onset of rotation. These textures evolve with time and arise from domains of different LC twist above and below the platelet. The domains eventually merge into a single one, giving the platelet a more uniform appearance (see Supplementary Fig. 5). The far-field director and polarization of the blue excitation light are shown by green and blue double arrows; parallel and crossed polarizers of the microscope are depicted by white double arrows. Scale bar is 5 µm. The movie is shown in real time.

File Name: Supplementary Movie 2
Description: Polarizing optical textures showing boojum stick-slip motion during the platelet spinning under continuous exposure to blue excitation light with Pe ǁ n0. Boojums become well pronounced after the "synchronization" of domains with different amounts of twist happens (in about 300s) and can be seen to stick to the vertices and slip on the edges of the hexagonal platelet. Their motion results in the fine features of the angular rotation of the platelet with corresponding periodicities (Fig. 2d,e). The far-field director and polarization of the blue excitation light are shown by green and blue double arrows; parallel and crossed polarizers of the microscope are depicted by white double arrows. Scale bar is 5 µm. The movie is shown in real time.

File Name: Supplementary Movie 3
Description: Comparison of platelet spinning under blue excitation light with different linear polarizations. Polarizing optical textures and spinning dynamics in this LC colloidal system depend on the polarization of the blue excitation light. This movie shows such spinning motions at linear polarizations of the excitation light at θ≈45° and θ≈-60° with respect to n0, which are indicated by the blue double arrow (see Supplementary Figs. 6, 7 for detailed analysis). The farfield director and polarization of the blue excitation light are shown by green and blue double arrows; parallel and crossed polarizers of the microscope are depicted by white double arrows. Scale bar is 5 µm. The movie is shown in real time.

File Name: Supplementary Movie 4
Description: Angular oscillations of a platelet under blue excitation light. This movie shows how platelet can oscillate between different angular orientations at low intensity of excitation light in a cell with thickness d=7μm. The far-field director and polarization of the blue excitation light are shown by green and blue double arrows; parallel and crossed polarizers of the microscope are depicted by white double arrows. Scale bar is 5 µm. The movie is presented in real time.

File Name: Supplementary Movie 5
Description: Angular oscillation of a platelet under continuous exposure to white light. This movie was taken under crossed polarizers with a retardation plate in-between. Incident polarization of the white light was set to be along the far-field director. Slow axis of the retardation plate is shown by a yellow double arrow; crossed polarizers of the microscope are depicted by white double arrows. The far-field director is shown by a green double arrow. Scale bar is 5 µm. The movie is shown in real time.

File Name: Supplementary Movie 6
Description: Light-driven spinning of colloidal cogwheel-like platelets under continuous exposure to blue excitation light. The movie demonstrates that the same mechanism that drives the spinning of colloidal hexagonal platelets as demonstrated before can be extended to particles of other shapes. 5

This movie shows such a system where a cogwheel-like platelet spins upon exposure to linearly polarized blue light with linear polarization at θ≈45° with respect to n0. The far-field director and polarization of the blue excitation light are shown by green and blue double arrows; parallel and crossed polarizers of the microscope are depicted by white double arrows. Scale bar is 5 µm. The movie is shown in real time.

File Name: Supplementary Movie 7
Description: Light-driven spinning of colloidal self-assemblies under continuous exposure to blue excitation light. Three hexagonal platelets self-assembled into a colloidal super-structure which readily responds upon exposure to linearly polarized blue light. The linear polarization was set to be at θ≈-45° with respect to n0 as indicated by the blue double arrow. The far-field director is shown by the green double arrow; parallel and crossed polarizers of the microscope are depicted by white double arrows. Scale bar is 5 µm. The movie is shown in real time.

File Name: Supplementary Movie 8
Description: Translational displacement of a platelet accompanied by spinning under powered by light. The linear polarization of blue excitation light was set to be at θ≈45° with respect to n0, as indicated by the blue double arrow. The far-field director is shown by the green double arrow. Parallel polarizers of the microscope are depicted by white double arrows. Scale bar is 5 µm. The movie is shown in 3× speed.

File Name: Supplementary Movie 9
Description: Translational displacement of a platelet accompanied by angular oscillations of orientation under continuous exposure to white light. The movie shows that linearly polarized white light with polarization perpendicular to n0 can also prompt translational displacement, accompanied by angular oscillations of orientation. The slow axis of the retardation plate is shown by a yellow double arrow whereas crossed polarizers of the microscope are depicted by white double arrows. The far-field director is shown by a green double arrow. The scale bar is 5 µm. The movie is shown in 3× speed.
